# Reaching out towards cannabis: approach-bias in heavy cannabis users predicts changes in cannabis use

**DOI:** 10.1111/j.1360-0443.2011.03475.x

**Published:** 2011-09

**Authors:** Janna Cousijn, Anna E Goudriaan, Reinout W Wiers

**Affiliations:** 1 ADAPT-lab, Department of Psychology, University of AmsterdamAmsterdam, the Netherlands; 2 Academic Medical Center, Department of Psychiatry, University of AmsterdamAmsterdam, the Netherlands; 3 Amsterdam Institute for Addiction ResearchAmsterdam, the Netherlands

**Keywords:** Approach avoidance task, approach-bias, cannabis, cannabis use disorder, craving, dependence

## Abstract

**Aims:**

Repeated drug exposure can lead to an approach-bias, i.e. the relatively automatically triggered tendencies to approach rather that avoid drug-related stimuli. Our main aim was to study this approach-bias in heavy cannabis users with the newly developed cannabis Approach Avoidance Task (cannabis-AAT) and to investigate the predictive relationship between an approach-bias for cannabis-related materials and levels of cannabis use, craving, and the course of cannabis use.

**Design, settings and participants:**

Cross-sectional assessment and six-month follow-up in 32 heavy cannabis users and 39 non-using controls.

**Measurements:**

Approach and avoidance action-tendencies towards cannabis and neutral images were assessed with the cannabis AAT. During the AAT, participants pulled or pushed a joystick in response to image orientation. To generate additional sense of approach or avoidance, pulling the joystick increased picture size while pushing decreased it. Craving was measured pre- and post-test with the multi-factorial Marijuana Craving Questionnaire (MCQ). Cannabis use frequencies and levels of dependence were measured at baseline and after a six-month follow-up.

**Findings:**

Heavy cannabis users demonstrated an approach-bias for cannabis images, as compared to controls. The approach-bias predicted changes in cannabis use at six-month follow-up. The pre-test MCQ emotionality and expectancy factor were associated negatively with the approach-bias. No effects were found on levels of cannabis dependence.

**Conclusions:**

Heavy cannabis users with a strong approach-bias for cannabis are more likely to increase their cannabis use. This approach-bias could be used as a predictor of the course of cannabis use to identify individuals at risk from increasing cannabis use.

## INTRODUCTION

Cannabis is the most commonly used illegal drug in most countries and treatment demands have increased strongly over the last decades [1–3]. Growing awareness of the addictive properties of cannabis is accompanied by a growing need for research investigating cannabis abuse and dependence. A key question in research on addiction is why some individuals escalate from recreational use to problematic use, while others do not. From all heavy cannabis users (defined as using cannabis on at least 10 occasions per month), an estimated 7–8% meet DSM-IV criteria of dependence [1–3]. Identifying predictors of the course of cannabis use is crucial for the development of effective prevention strategies.

Inability to control drug use is considered a core aspect of drug dependence [4–8]. Despite awareness of harmful consequences, an addicted person compulsively continues to use drugs, with frequent failed attempts to cut down or quit. These addictive behaviours have been hypothesized to arise from an imbalance between an approach-orientated motivational system and a regulatory executive system [4–8]. After repeated drug exposure, the motivational system becomes sensitized towards a drug, which can lead to relatively automatically triggered tendency to approach drug-related stimuli [8,9]. A drug-orientated motivational system combined with insufficient executive resources (possibly compromised by drug use) promotes escalation of drug use.

Cognitive biases for drug-related cues are thought to be behavioural manifestations of a drug-orientated motivational system. An abundance of studies, focusing mainly on alcohol and tobacco use, highlight roles of attentional, approach and evaluative biases in the development and maintenance of addictive behaviours ([10–18]; for reviews see [19–21]). In drug-abusing and -dependent individuals compared to non-dependent individuals, drug-related cues automatically capture attention, evoke approach tendencies and are evaluated as more positive and arousing in comparison to neutral cues. Recently, evidence has emerged that these processes are also present in heavy cannabis users. Unlike controls, heavy cannabis users are biased in detecting subtle cannabis-related changes in complex scenes [22,23], implying higher attention to cannabis than neutral cues. Further, in heavy cannabis users, an attentional bias for cannabis-related words is associated with craving, frequency of use and severity of dependence [24,25]. Compared to non-users, heavy cannabis users maintain their gaze longer upon cannabis cues, are faster in approaching cannabis cues and rate cannabis cues as more pleasant compared to neutral cues [26]. Finally, in adolescents at risk for drug abuse, evaluative biases, measured with a word association task and Implicit Association Task (IAT), were found to predict cannabis use [27].

The cognitive bias central in this study is an approach-bias for cannabis-related stimuli. Tasks used previously to assess an approach-bias for substance-related stimuli are the Stimulus Response Compatibility Task (SRC [10,12,13,15,26]), IAT [16,17,28] and Approach Avoidance Task (AAT [9,29,30]). These tasks differ on a number of dimensions. First, in both the SRC and IAT, participants are instructed explicitly to categorize target stimuli (e.g. substance-related or neutral) with ‘approach’ in one block and ‘avoid’ in another block of the task (in the SRC, but not the IAT, this is accompanied by a symbolic approach or avoidance movement of a manikin). The approach-bias is then derived from reaction-time (RT) differences between these two categorizations. In contrast, the AAT measures approach and avoid action tendencies by asking participants to pull or push a joystick in response to a content-irrelevant feature; for example, format or orientation of images [9,29,30]. The AAT also uses a ‘zooming feature’: pulling the joystick increases the picture's size while pushing decreases it, which in itself already generates the sense of approach or avoidance, respectively. Combining this zooming feature with actual arm flexion and extension results in more realistic approach and avoid actions, compared to the SRC and IAT (for a comparison between SRC and AAT see [31]). Further, responding to a feature irrelevant to the content might be more likely to tap into automatic motivational processes [32].

Regarding the approach-bias in cannabis users, to the best of our knowledge only one SRC study exists, which showed faster (biased) approach responses to cannabis cues in heavy cannabis users compared to non-users [26]. The aim of the present study was to investigate the approach-bias in cannabis users with the AAT, and to investigate the predictive relationship between the approach-bias and levels of cannabis use, craving and the course of cannabis use over 6 months. We hypothesized that cannabis users would show a stronger approach-bias towards cannabis-related images compared to controls. We expected that a stronger approach-bias in heavy cannabis users would be related to higher levels of craving and cannabis use and problems. We also expected that heavy cannabis users with larger approach-biases would be more likely to increase cannabis use and levels of dependence after 6 months.

## METHOD

### Participants

Thirty-two heavy cannabis users and 41 controls aged 18–25 were recruited through advertisements on the internet and in cannabis outlets (coffee-shops). Groups were matched for age, gender and estimated intelligence [33,34] (Table 1). Heavy cannabis use was defined as using cannabis on 10 or more days in the last month, on at least 240 days in the last 2 years, and not seeking treatment or having a history of treatment for cannabis use [35,36]. Participants in the control group used cannabis on fewer than 50 life-time occasions and did not use last year [36]. Drug and alcohol use was controlled for by excluding participants with an Alcohol Use Disorder Identification Test (AUDIT) score higher than 10 [37], smoking more than 20 cigarettes daily or using non-cannabinoid drugs on more than 100 occasions [35,36]. Other exclusion criteria were a history of major medical, physical or psychiatric disorders, which was assessed with the Mini-International Neuropsychiatric Interview (MINI [38], Dutch version 5.0.0). The medical ethics committee of the Academic Medical Centre approved the study and all participants signed informed consent before participation.

**Table 1:** Sample characteristics at baseline and 6-month follow-up

	*Heavy cannabis users*		*Controls*	
		
	*Baseline*	*Six-month follow-up*	*Baseline*	*Six-month follow-up*
*n* (% female)	32 (34)	30 (33)	41 (37)	41 (37)
Age, mean (SD)	21.2 (2.4)	21.7 (2.4)	22.0 (2.5)	22.5 (2.5)
Verbal IQ (Dutch reading test), mean (SD)	104.1 (5.5)	–	105.2 (6.8)	–
Alcohol use and related problems (AUDIT), mean (SD)	6.2 (3.3)	5.6 (3.2)	4.9 (3.0)	4.8 (3.2)
Cigarette smoking (%)	69	63	17**	20**
Cigarette dependence (FTND)	2.8 (2.4)	2.9 (2.5)	0.5 (1.1)	0.6 (1.2)
Cannabis use life-time (episodes), mean (SD)	1607.1 (1438.9)	1622.5 (1349.1)	4.4 (9.1)	5.0 (10.0)
Cannabis use and related problems (CUDIT), mean (SD)	12.4 (5.8)	9.5 (6.6)*	0 (0)	0.2 (0.5)
Cannabis dependence (DSM-IV criteria-count)	2.3 (1.6)	1.5 (1.5)*	0 (0)	0 (0)
Duration heavy cannabis use (year), mean (SD)	2.5 (1.9)	2.9 (1.9)	–	–
Cannabis use days/week, mean (SD)	4.9 (1.5)	4.9 (2.1)	–	–
Cannabis use g/week, mean (SD)	3.0 (2.2)	3.2 (3.0)	–	–

Craving (baseline)	Pre-test	Post-test	Pre-test	Post-test
MCQ compulsivity	6.6 (4.1)	7.9 (4.3)	3.0 (0.3)	3.1 (0.3)
MCQ emotionality	5.9 (3.8)	7.0 (3.9)	3.2 (0.6)	3.3 (1.2)
MCQ expectancy	9.3 (3.9)	10.0 (3.4)	3.3 (0.8)	3.3 (0.9)
MCQ purposefulness	8.9 (3.9)	12.5 (5.1)***	3.1 (0.3)	3.2 (0.1)

* *P* < 0.05 for baseline follow-up comparison

** *P* < 0.001 for group comparison

*** *P* < 0.001 for pre-test–post-test comparison

SD: standard error; MCQ: marijuana craving questionnaire; AUDIT: alcohol use disorder identification test; CUDIT: cannabis use disorder identification test; FTND: Fagerström Test for Nicotine Dependence.

### Cannabis use, tobacco use and craving

Cannabis use and related problems were assessed with the Cannabis Use Disorder Identification Test (CUDIT [39]) and a structured diagnostic interview (MINI [38]). The CUDIT is a screening instrument for at-risk cannabis use and consists of 10 items on cannabis use frequencies, symptoms of dependence and use-related problems [39,40]. From the diagnostic interview, the DSM-IV criteria-count for cannabis dependence was used for analysis. In addition, history of past and present cannabis use was obtained (e.g. life-time episodes, duration of heavy use, days per week, weekly use in grams, ways of using). Tobacco use and dependence were measured with the Fagerström Test for Nicotine Dependence (FTND [41]). The short version of the Marijuana Craving Questionnaire (MCQ) was used to assess craving before (pre-test) and after test-session (post-test) [42]. The MCQ is reliable for assessing craving in cannabis users not seeking treatment [42,43]. Items were rated on a Likert response scale ranging from 1 (strongly disagree) to 7 (strongly agree). It distinguishes four three-item craving factors: compulsivity (inability to control use, e.g. ‘I need to smoke marijuana now’), emotionality (relief from withdrawal and negative affect, e.g. ‘I would feel less anxious if I smoked marijuana right now’), expectancy (anticipation of positive outcomes, e.g. ‘smoking marijuana would make me content’) and purposefulness (planning/intention to use for positive outcomes, e.g. ‘smoking marijuana would be pleasant right now’). Craving scores for each factor were obtained by summing item scales. Pre-test, post-test and pre-test–post-test difference scores were used as independent variables.

### Approach avoidance task

**Figure 1:**
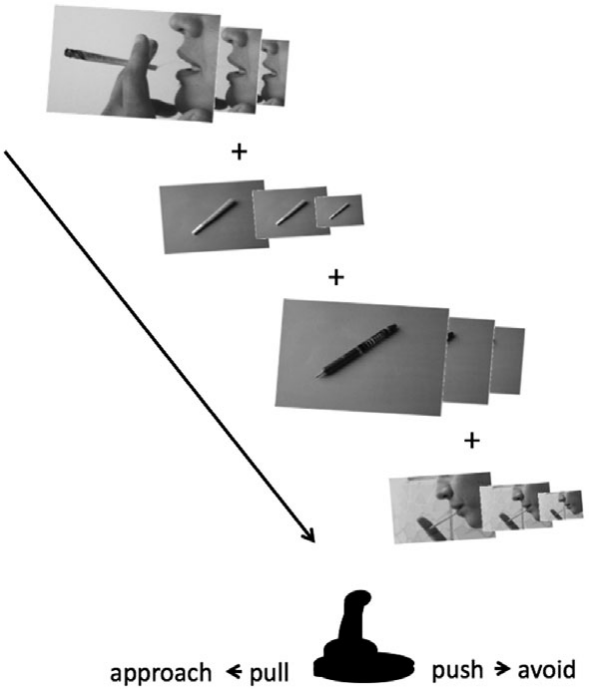
Schematic representation of the Approach Avoidance Task

An adapted AAT [9,29,30] was used to measure biases in automatic action-tendencies towards cannabis (see Fig. 1). Participants viewed 20 cannabis-related images and 20 neutral images. Cannabis-related images were close-ups of cannabis, objects for using cannabis and individuals smoking cannabis. Neutral images were close-ups of individuals and objects matched visually to the cannabis-related images on colour and composition. All images were rotated 3° left or right. Image content was irrelevant to the task: participants were instructed to pull or push a joystick in response to rotation direction. Half the participants pushed images rotated left and pulled images rotated right, while the other half received opposite instructions. Pulling and pushing the joystick gradually increased and decreased image-size. This zooming feature combined with arm flexion and extension mimics approach and avoid actions [9,29,30]. Each image was presented four times, twice in push- and twice in pull-format. The resulting 160 trials were presented in semi-random order (at most three similar rotations and image categories in a row) and preceded by 15 practice trials with grey rectangles.

### Procedure

Test-sessions took place during the late afternoon and at the beginning of the evening. All participants were asked to refrain from alcohol and drug use 24 hours prior to testing. Each session started and ended with filling out the MCQ. After completing other questionnaires, administering cannabis use history and the diagnostic interview, participants performed the AAT. Six months later participants were contacted for a telephone interview on present drug use and related problems.

### Data preparation and statistical analysis

To correct for outliers, RTs below 200 ms, above 2000 ms and more than 3 standard deviations (SD) above and below the mean were removed for each participant. Error trials were removed. The bias score was calculated by subtracting median approach RT from median avoid RT for each image category. The subtraction resulted in a bias score for cannabis images and neutral images for each participant. As in previous AAT publications, median RTs were used because they are less sensitive to outliers than means [9,29,30,44]. A positive score indicated a relatively faster approach compared to avoid RTs, whereas a negative score indicated a relatively faster avoid compared to approach RTs for a given image category. A positive or negative bias score will be referred to further as an approach-bias or avoid-bias. To validate the AAT, Cronbach's alpha was calculated for the cannabis and neutral condition with the separate bias scores for each image. Internal reliability of the cannabis bias score (Cronbach's α = 0.68) and neutral bias score (Cronbach's α = 0.61) was acceptable. To compare groups, AAT bias scores were analysed using standard analysis of variance (ANOVA). One-sample *t*-tests were used to test if bias scores deviated significantly from zero within each group. Pearson's correlations and sequential multiple regression analyses were used to investigate associations between AAT bias scores, craving, measures of cannabis use and related problems at baseline and 6-month follow-up and tobacco smoking. To control for general biases in approach and avoid action-tendencies, partial correlations and regression analysis were conducted with neutral bias score as covariate.

## RESULTS

### Sample characteristics

#### Baseline

One control was discarded as outlier because bias scores were above 6 SD from the mean (group comparison was still significant including this participant). Due to technical error, data from another control was lost. The remaining 71 participants' scores were 95% correct (range = 78–100), with no differences between groups. The groups did not differ in age (*t*_71_ = 1.30, *P* = 0.20), gender (χ^2^ = 0.00, *P* = 1.00), IQ (*t*_71_ = 0.76, *P* = 0.48) and alcohol use (*t*_71_ = 1.36, *P* = 0.18), but there were more tobacco smokers among heavy cannabis users (χ^2^ = 22.26, *P* < 0.001; see Table 1). Overall median RTs did not differ between groups (*t*_69_ = 0.88, *P* = 0.38). In heavy cannabis users, post-test craving was higher for the MCQ purposefulness factor (*t*_31_ = 5.3, *P* < 0.001) (see Table 1).

#### Six-month follow-up

A 97% follow-up rate was achieved after 6 months (two non-responders among the heavy cannabis users). Within heavy cannabis users, Little's Missing Completely At Random (MCAR [45]) test with all study variables indicated that the two non-responders were missing at random (χ^2^ = 20.3, d.f. = 20, *P* = 0.44). Average DSM-IV criteria-count (*t*_29_ = 2.3, *P* = 0.026) and CUDIT-scores (*t*_29_ = 2.2, *P* = 0.035) decreased in heavy cannabis users (see Table 1). Cannabis use frequencies and measures of alcohol and tobacco use did not change in both heavy cannabis users and controls (see Table 1).

### AAT bias scores in heavy cannabis users and controls

#### Group comparison

**Figure 2:**
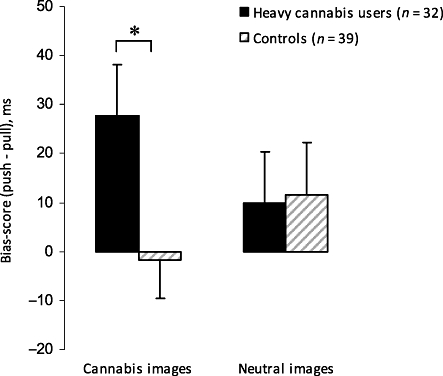
Mean Approach Avoidance Task (AAT) bias score for cannabis and neutral images in heavy cannabis users and controls (ms with standard error bars). A positive score indicates faster reaction times on pull (approach) trials compared to push (avoid) trials. Heavy cannabis users have an approach-bias towards cannabis-related images and their bias score significantly differs from controls (*P < 0.05)

Differences in AAT bias scores were first analysed with a mixed ANOVA with group as between-subject factor and image type as within-subject factor with two levels (cannabis and neutral images). Homogeneity of variance assumption was not violated (*P* > 0.12). There was no main effect of image type (*F*_1,69_ = 0.09, *P* = 0.77). The interaction between image type and group was significant (*F*_1,69_ = 4.53, *P* = 0.037, η^2^ = 0.062). In line with our hypothesis, heavy cannabis users had larger cannabis bias scores than controls (*t*_69_ = 2.33, *P* = 0.023, *d* = 0.55), whereas neutral bias scores did not differ between groups (*t*_69_ = −0.12, *P* = 0.91). Figure 2 shows mean bias score per image type for each group. Heavy cannabis users were faster in approaching compared to avoiding cannabis images because the cannabis bias score was larger than zero (*t*_31_ = 2.64, *P* = 0.013, *d* = 0.47). This cannabis bias score reflected an approach-bias: approach RTs for cannabis (mean = 803.7, SD = 80.1) were faster than for neutral images (mean = 827.5, SD = 79.7; *t*_31_ = 3.61, *P* < 0.001, *d* = 0.64), whereas avoid RTs for cannabis (mean = 831.4, SD = 72.5) and neutral images (mean = 837.3, SD = 75.1) did not differ (*t*_31_ = 0.63, *P* = 0.54). None of the other bias scores deviated from zero (*P* > 0.28).

#### Correlations

In contrast to our hypothesis, the approach-bias correlated negatively with craving in heavy cannabis users: higher approach-bias was related to lower pre-test scores on the MCQ emotionality (*R* = −0.36, *P* = 0.049) and expectancy factors (*R* = −0.36, *P* = 0.049), but not the compulsivity (*R* = 0.08, *P* = 0.68) and purposefulness factors (*R* = −0.24, *P* = 0.20). The approach-bias correlated positively with weekly cannabis use at 6-month follow-up: higher approach-bias was related to higher levels of cannabis use (in grams, *R* = 0.42, *P* = 0.023). The approach-bias did not correlate with post-test craving, baseline cannabis use, baseline and 6-month follow-up measures of cannabis use-related problems and tobacco smoking.

### Predictors of cannabis use at 6-month follow-up in heavy cannabis users

To assess the predictive relationship between approach-bias and changes in weekly cannabis use after 6 months (in grams) in heavy users, hierarchical multiple regression was performed. To control for other variables, baseline weekly cannabis use (in grams), CUDIT score, DSM-IV criteria-count, craving factors (pre–post-test average) and neutral bias score were entered first, after which the approach-bias was entered. Preliminary analyses indicated no violation of the assumption of normality, linearity, multicollinearity and homoscedasticity (maximum Cook's distance = 0.63, maximum standardized residual = 2.0). The total variance explained by the final model was 76% (*F*_9,20_ = 7.00, *P* < 0.001; table 2). The control variables explained 63% of the variance in cannabis use 6 months later. Baseline weekly cannabis use (*P* < 0.001) was a significant predictor in the first step (compulsive craving was marginally significant, *P* = 0.053). The approach-bias explained an additional 13% of the variance in cannabis use 6 months later (*F* change_1, 20_ = 10.65, *P* = 0.004). Participants with a stronger bias to approach cannabis used more cannabis at 6-month follow-up. Besides the approach-bias, only baseline weekly cannabis use was a significant predictor in the final model. In contrast to our hypothesis, no predictive relations were found between the approach-bias and changes in measures of cannabis-related problems and dependence (CUDIT score, DSM-IV criteria count) after 6 months.

**Table 2:** Hierarchical multiple regression analysis for variables predicting weekly cannabis use (in grams) at 6-month follow-up in heavy cannabis users (*n* = 30)

	*B*	*SE B*	β
**Step 1:** change *R*^2^: 0.63**			
Weekly cannabis use baseline	1.05	0.28	0.61**
CUDIT	−0.07	0.10	−0.14
DSM-IV criteria-count	0.57	0.33	0.32
MCQ compulsivity	0.46	0.23	0.55
MCQ emotionality	−0.29	0.21	−0.32
MCQ expectancy	0.03	0.23	0.03
MCQ purposefulness	−0.06	0.16	−0.08
AAT neutral bias score	0.00	0.01	0.03
**Step 2:** change *R*^2^: 0.13*			
Weekly cannabis use baseline	1.09	0.23	0.63**
CUDIT	−0.01	0.09	−0.01
DSM-IV criteria-count	0.43	0.27	0.24
MCQ compulsivity	0.21	0.20	0.25
MCQ emotionality	0.02	0.20	−0.04
MCQ expectancy	0.01	0.17	0.01
MCQ purposefulness	−0.04	0.14	−0.05
AAT neutral bias score	−0.01	0.01	−0.23
AAT cannabis bias score	0.03	0.01	0.53*

* *P* < 0.01

** *P* < 0.001.

Final model *R*^2^: 0.76**, adjusted *R*^2^ 0.65. SE: standard error; MCQ: marijuana craving questionnaire; CUDIT: cannabis use disorder identification test; AAT: approach audience task.

## DISCUSSION

This study showed that heavy cannabis users, but not controls, have an approach-bias specifically for cannabis-related images (not for neutral images), as measured with the AAT [9,29,30]. In line with our hypothesis, the approach-bias predicted changes in cannabis use 6 months later in heavy cannabis users: stronger approach-biases were related to increases in weekly cannabis use. In contrast to our hypothesis, the approach-bias was related negatively to craving for relief from negative affect and anticipation of positive outcome (i.e. MCQ emotionality and expectancy factor). No associations were found between the approach-bias and measures of cannabis-related problems and dependence.

The approach-bias found here in heavy cannabis users supports the idea that an approach-bias for substance-related stimuli is a common phenomenon in cannabis users as well as in alcohol users and tobacco smokers [9,10,12,15–17]. The most important finding is that the approach-bias predicted changes in cannabis use after 6 months. Heavy cannabis users with stronger approach-biases were more likely to increase weekly cannabis use, while lower approach (or even avoid) biases were related to decreases in use. To our knowledge, this study is the first to find a prospective predictive relation between an approach-bias and course of drug use. This predictive relationship may have clinical implications. Even after prolonged drug use, some heavy using individuals develop abuse and dependence while others do not. The approach-bias could be a predictor of the course of drug use. It might be used for identifying individuals especially at risk for increasing cannabis use for targeted interventions. An advantage of using implicit measures such as the AAT is that they do not rely upon self-report. Insight into severity of drug dependence and self-awareness might be compromised in dependent individuals, thereby influencing the reliability of self-reports [46]. A second clinical implication could be using a modified AAT to retrain heavy cannabis users to avoid cannabis. Recent studies showed that approach action tendencies in heavy alcohol drinkers and alcohol-dependent patients can be modified [30,47]. Successful training to avoid alcohol was related to decreased subsequent alcohol use and improved treatment outcome. Future research is needed to verify this in heavy cannabis users and clinical cannabis users, as has been shown recently for alcohol-dependent patients [47].

In contrast to our hypothesis, no associations were found between approach-bias and changes in measures of cannabis dependence. This could be due to methodological issues. Inherent to the sample, cannabis-related problems were relatively low and a 6-month follow-up might have been too short to detect changes in measures of dependence. An alternative explanation is that cognitive biases such as the approach-bias mainly play a role in the course of drug use in the *earlier* stages of addiction. The approach-bias may predict who will use more, but not who will progress to problematic drug use. This appears to disagree with the incentive sensitization theory of addiction [48,49], and seems more in line with theories where incentive sensitization is mainly important during escalation of drug use and less when subsequent compulsive drug use progresses [50,51]. To test this hypothesis, associations between approach-bias and prospective cannabis use needs to be assessed in larger samples of dependent, heavy and sporadic cannabis users compared to non-using controls.

Also in contrast to our hypothesis, the approach-bias was associated negatively with pre-test levels of craving for relief from negative affect and craving for anticipation of positive outcome. Post-test craving was not associated with the approach-bias. Compulsive craving predicted cannabis use after 6 months: higher craving was related to increased use. However, the effect disappeared when the approach-bias entered the regression model. Most theories predict a bidirectional positive association between approach-biases and craving [6–8]. A recent meta-analysis showed weak positive relations between craving and attentional bias for alcohol [52]. In cannabis users a positive relation between post-test craving and attentional bias has been reported [25], although no relationships between post-test craving, attentional bias and approach-bias were found in a different study [26]. Clearly, more research is needed to assess relationships between cognitive biases and craving. Further, the MCQ can differentiate reliably between craving factors [42], but a theoretical framework should be developed further, which is beyond the scope of this paper. However, our findings emphasize the relevance of measuring both pre- and post-test craving and using factorial decomposition of self-reported craving.

Finally, some limitations must be taken into account. First, there were more tobacco smokers among heavy cannabis users compared to controls, and almost all cannabis users smoked cannabis cigarettes combined with tobacco (most common use-form in the Netherlands [53]). Tobacco might increase the effects of cannabis [54], and the resemblance between tobacco and cannabis cigarettes possibly activates approach actions towards tobacco in tobacco users. Neither in heavy cannabis users nor in controls was tobacco use associated with the approach-bias. However, our sample prevents discrimination between cannabis and tobacco effects. Secondly, in the course towards dependence, increased sensitivity to general rewards might precede incentive salience of drugs over natural rewards [5]. Indeed, it has been reported that heavy drinking male carriers of the OPRM1 G-allele had an approach-bias for both alcohol and other appetitive stimuli [9]. However, with the present design it cannot be determined if the approach-bias in heavy cannabis users generalizes to other rewarding stimuli. Thirdly, the results should be interpreted bearing in mind that the approach-bias reflects the relative difference between approaching and avoiding cannabis images. Although the group comparison suggests that strong approach tendencies for cannabis, rather than weak avoid tendencies for cannabis, predict changes in prospective cannabis use in heavy users, the present findings with a relative measure are not conclusive regarding this issue. Alternatively, the interplay or conflict between approach and avoid tendencies may predict changes in cannabis use. This is an important question that needs to be addressed in future research. Fourthly, the AAT is a relatively new measure and its temporal stability is unknown. Finally, the absence of a relation between approach-bias and baseline levels of cannabis use might suggest a limitation in the construct validity of the task.

In conclusion, heavy cannabis-smoking young adults automatically activate approach action tendencies in response to cannabis-related stimuli (approach-bias), and the extent to which they do so predicts further escalation of their use.
